# Paramagnetic contrast medium in high-level athletes with lower limb muscle injuries: can it make the return to sport safer reducing the recurrence rate?

**DOI:** 10.1007/s11547-022-01472-x

**Published:** 2022-03-14

**Authors:** Marco Calvi, Marco Curti, Stefano Mazzoni, Lucio Genesio, Rodolfo Tavana, Leonardo Callegari, Eugenio Annibale Genovese

**Affiliations:** 1 Department of Diagnostic and Interventional Radiology, University of Insubria, 21100 Varese, Italy; 2 Team Physician Milan A.C., Milano, Italy; 3 Team Physician Torino F.C., Torino, Italy; 4 Department of Diagnostic and Interventional Radiology, ASST-Settelaghi, Ospedale di Circolo e Fondazione Macchi, 21100 Varese, Italy; 5 Medical Clinical Institute Intermedica - Columbus, via Buonarroti 48, 20145 Milan, MI Italy

**Keywords:** Athletes, Lower extremity, Magnetic resonance imaging, Muscles, Reinjuries

## Abstract

**Purpose:**

The aim is to investigate whether contrast medium can improve accuracy in the assessment of healing muscle injury in high-level professional athletes.

**Materials and methods:**

Our series is a retrospective study including the records of 22 players (mean age 28 ± 5 SD) with lower limbs muscle injuries type 3a (Mueller-Wohlfarth). All athletes received two MRIs: the day after the injury and before resuming heavy effort activities. Contrast medium uptake was measured in the second MRI by comparing the mean enhancement at the lesion site (ME) with that of the healthy contralateral muscle (HM). The result is a percentage referred to as muscular contrast index (MC index). The difference between the mean MC index value between athletes with and without re-injury was assessed with both the Mann–Whitney and the Kruskal–Wallis test.

**Results:**

Twenty-nine muscle injuries matched the inclusion criteria. The mean MC index values, adjusted for the variable of time elapsed between the last contrast examination and return to the field, were significantly different in the two study groups (*p* < .001).

**Conclusion:**

The contrast medium in the follow-up of muscle injuries may be useful in determining the degree of scar stability in a healing injury. Injuries with a high MC index were found to be ‘unstable’, with a higher rate of recurrence than those with a low MC index. Resumption of competitive activity after achieving not only clinical resolution but also a satisfactory MC index value may increase the safety of return to the field and reduce the recurrence rate.

## Introduction

- The paramagnetic contrast medium is useful in differentiating mature from unstable scar tissue.
- The clinician can use the MC index as an extra tool in planning the athlete's return to sport.
- Clinical impact in elite athletes results in optimised recovery times.
- Diagnosing ‘unstable’ lesions would result in a lower recurrence rate.

## Key points

Muscle tears are a common issue for professional athletes, being more than 30% of all injuries in elite football, burdened by prolonged periods of rest from competition associated with strong external pressure for a rapid return to sport (RTS) [1, 2]. Inaccurate estimation of injury grade and, so, incorrect prognosis, may lead to premature RTS with an increased risk of re-injury [3, 4].

The purpose of a correct assessment of the extent of the muscle injury correlates with the possibility of a safe and prompt return thus missing as few matches as possible.

Over time, many classifications have been proposed with the aim of supplying a prognostic tool in the management of muscle injuries. The first were based on clinical criteria (i.e. the American Medical Association (AMA) classification) [2], followed later by classifications based on imaging criteria. The latter include, for example, the Peetrons’ classification [5], based on ultrasound findings and the Chan [6] and Cohen’s [4] classifications based on magnetic resonance imaging [2]. MRI-based classifications have evaluated several variables including correlation with time of injury to provide a prognostic tool that could aid the clinician in the management of an athlete [2]. However, the results obtained were sometimes contradictory and many conclusions still need true clinical validation [2, 5, 7, 8].

These grading systems had the problem of grouping of injuries into broad categories thereby impeding the correlation of the degree of the lesions with its different aetiology, treatment pathway and prognostic relevance [9–11]. In 2012, Mueller-Wohlfarth et al. designed a classification to standardise the many different classifications and to ease diagnostic, therapeutic and scientific communication [12].

This comprehensive and practical classification system was introduced to classify the broad spectrum of muscle injuries more precisely (Table 1).

**Table 1:** Mueller-Wohlfahrt classification

A. Indirect muscle disorder/injury Muscle disorder	Functional muscle disorder	Type 1: Overexertion-related muscle disorder	Type 1A: Fatigue-induced muscle disorder Type 1B: Delayed-onset muscle soreness (DOMS)
		Type 2: Neuromuscular muscle disorder	Type 2A: Spine-related neuromuscular Muscle disorder Type 2B: Muscle-related neuromuscular
	Structural muscle injury	Type 3: Partial muscle tear	Type 3A: Minor partial muscle tear Type 3B: Moderate partial muscle tear
		Type 4: (Sub)total tear	Subtotal or complete muscle tear Tendinous avulsion
B. Direct muscle injury		Contusion/Laceration	

Mueller-Wohlfahrt classification summary [12]

Based on this classification, before the resumption of sport activity we introduced the paramagnetic contrast medium in the imaging control protocol of ‘minor partial muscle tear’ (3a according to the Munich classification system) [12]. During post-processing, we analysed the difference in enhancement between the muscle injury and the contralateral healthy area trying to predict the remaining injury time.

The aim of our study is to evaluate the utility of paramagnetic contrast medium in monitoring a healing muscle injury to discriminate stable from unstable scars to reduce the risk of re-injury and to define the prognosis more accurately for RTS (371/400).

## Materials and methods

### Patient and public involvement statement

This is a retrospective study based on existing clinical data. Patients were not directly involved, provided that written informed consent for contrast-enhanced MRI was obtained from each of them.

Patients also signed a comprehensive consent form which satisfied all the requirements of the Declaration of Helsinki and the Italian national law for the protection of personal data.

### Participants

Between February 2012 and February 2021, 260 MRI studies of high-level professional athletes with clinically suspected lower limb muscles injuries were selected. Each athlete complained of acute pain in the lower limb with indirect trauma during physical activity; the clinical suspicion of a muscle lesion was confirmed by the club physician.

From the initial sample, we selected the athletes who received at least two MRIs with the following characteristics: the first MRI performed immediately after the injury; the second one before resuming heavy effort activities after complete resolution of symptoms. MRIs specific protocols will be discussed in the following sections.

In some cases, paramagnetic contrast medium was used to assess the healing status of the lesions. Only athletes who had at least one contrast-enhanced MRI scan before resuming training were included in the study. Finally, only type 3a injury according to Mueller-Wohlfarth’s classification was included [12].

Regarding injuries occurring at the end of the season, sometimes it happened that no real return to sport date was available. In this study, only injuries with complete data were included, leaving out those occurring at season's end.

Examinations of athletes with chronic or recurrent pain or with contraindications to MRI or contrast medium administration use were excluded.

Urgent need for surgery, complete avulsion injuries, presence of concomitant fracture or presence of double lesions were considered exclusion criteria.

### Images’ evaluation

A radiologist (EAG), with more than 20 years of experience in musculoskeletal radiology, interpreted each MRI study classifying the abnormal findings according to the Mueller-Wohlfarth’s classification [12]. The same radiologist retrospectively evaluated all the studies, blinded to the original report, providing a second score. If signs of a higher-grade injury were present, the injury was classified according to the highest grade. During the second evaluation, a simple and reproducible method for measuring enhancement in contrast medium MRIs was introduced.

We used the Cohen test to measure the degree of intra-operator diagnostic reliability between the first and second evaluation.

### Muscle contrast index (MC index)

The measurement method required the use of contrast-enhanced T1 axial sequences and consisted of drawing two approximately circular ROI (about 10 mm^2^ ± 2mm^2^ depending on the lesion size and location), the first in the region of maximum visual enhancement (ME) where a lesion was detected, the second in the contralateral healthy muscle (HM) (both the ROIs were placed in the same section).

Each measurement was taken on the last contrast-enhanced MRI before resuming training, choosing the most representative anatomical region where the lesion was mostly clear on the first MRI after the injury. The anatomical region was chosen using axial DWI and DP fat-sat-weighted sequences.

Measurements were performed taking care not to include vessels, fluid collections or fat tissue in the measurement area.

The average value of the signal intensity at the point of maximum enhancement was then subtracted from the average intensity value of the healthy muscle. The resulting value was then divided by the HM.

The result was expressed as a percentage rise in enhancement of the injured tissue compared to the healthy muscle and was called muscle contrast index (MC index) = (ME − HM)/HM * 100.

### MRI parameters

All images were obtained using a 1.5-Tesla magnet system (Philips Ingenia Ambition/Elition, Philips Medical System, Eindhoven, Netherlands) with a phased array 16 channels body matrix coil.

The first MRI after the injury was performed according to the protocol illustrated in Table 2(a).

**Table 2:** MRI technique

(a) First study without contrast medium	(b) Contrast-enhanced studies
Coronal STIR TSE (TR 2700–6000, TE 90, TI 140 ms, FOV 400–450 × 400, Slice thickness 4 mm, Matrix 328 × 310, ETL 6, TURBO FACTOR 20, NSA 2, SENSE reduction factor 2)	Coronal STIR TSE (TR 2700–6000, TE 90, TI 140 ms, FOV 400–450 × 400, Slice thickness 4 mm, Matrix 328 × 310, ETL 6, TURBO FACTOR 20, NSA 2, SENSE reduction factor 2)
Axial TSE dual proton density-weighted SPAIR and without fat suppression (TR 3000–4000, TE-1 5.7 ms, TE-2 80 ms, FOV 400X300, Slice thickness 4 mm, Matrix 400 × 250, TURBO FACTOR 18, NSA 2, SENSE reduction factor 2)	Axial TSE dual proton density-weighted SPAIR (TR 3000–4000, TE1 5.7 ms, TE2 80 ms, FOV 400X300, Slice thickness 4 mm, Matrix 400 × 250, TURBO FACTOR 18, NSA 2, SENSE reduction factor 2)
Axial T1 TSE (TR 520, TE 18, FOV 400X300, Slice thickness 4 mm, Matrix 400 × 250, TURBO FACTOR 5, NSA 2, SENSE reduction factor 2); Axial DWI (b = 0–450-900) (TR 1759, TE 80–90, FOV 450X400, Slice thickness 4 mm, Matrix 152 × 133, NSA 4, SENSE reduction factor 2)	Axial contrast-enhanced T1 TSE (TR 520, TE 18, FOV 400X300, Slice thickness 4 mm, Matrix 400 × 250, TURBO FACTOR 5, NSA 2, SENSE reduction factor 2)
Axial DWI (b = 0–450–900)(TR 1759, TE 80–90, FOV 450X400, Slice thickness 4 mm, Matrix 152 × 133, NSA 4, SENSE reduction factor 2)	Axial DWI (b = 0–450–900)(TR 1759, TE 80–90, FOV 450X400, Slice thickness 4 mm, Matrix 152 × 133, NSA 4, SENSE reduction factor 2)

The technical protocol of follow-up requires contrast medium injections (from 10 to 15 ml of Dotarem—Guerbet, France—according to the athlete's weight, 0.2 ml/kg) and was performed as illustrated in Table 2(b). T1 axial images acquisition started immediately after contrast medium administration.

### Treatment and time to return to sport (RTS)

Athletes included in the prospective case series received either a similar rehabilitation programme or individualised rehabilitation at the club or federation.

Time to RTS was defined as the number of days from injury until the athlete was cleared to resume unrestricted training by the treating physician or physiotherapist at the club or federation. The treating physician or physiotherapist making the RTS decision was not blinded to the MRI findings.

The number of days until RTS was provided by the club medical staff. After the resumption of sporting activity, a period of one month was considered to detect any re-injury.

The term re-injury refers to the occurrence of a new injury where the old injury was forming reparative tissue that would eventually evolve into a stable scar.

The athletes were divided into two groups according to the presence of a re-injury and, for each of them, the total injury time and the time between the last contrast medium examination and return to the field were calculated.

### Statistical analyses

The difference between the average MC index in patients without and with re-injury was calculated. Both data sets were tested for normality and presented as mean (± standard deviation; SD). The Student's *t* test was applied in cases where all criteria were met. In all other cases, the Mann–Whitney and the Kruskal–Wallis tests were applied.

Categorical data were presented as frequency (%). The results were accepted as significant at *p* < 0.01.

The value of each MC index was adjusted to the time between the date of the last examination and the actual return to the field. The rationale for this choice was to eliminate the variable of time between the last contrast examination and the actual return to the field.

The data analysis for this paper was generated using the Real Statistics Resource Pack software (Release 7.2); Copyright (2013–2020) Charles Zaiontz. www.real-statistics.com.

This paper is consistent with the STROBE cohort reporting guidelines [13].

## Results

### Patient characteristics

**Fig. 1:**
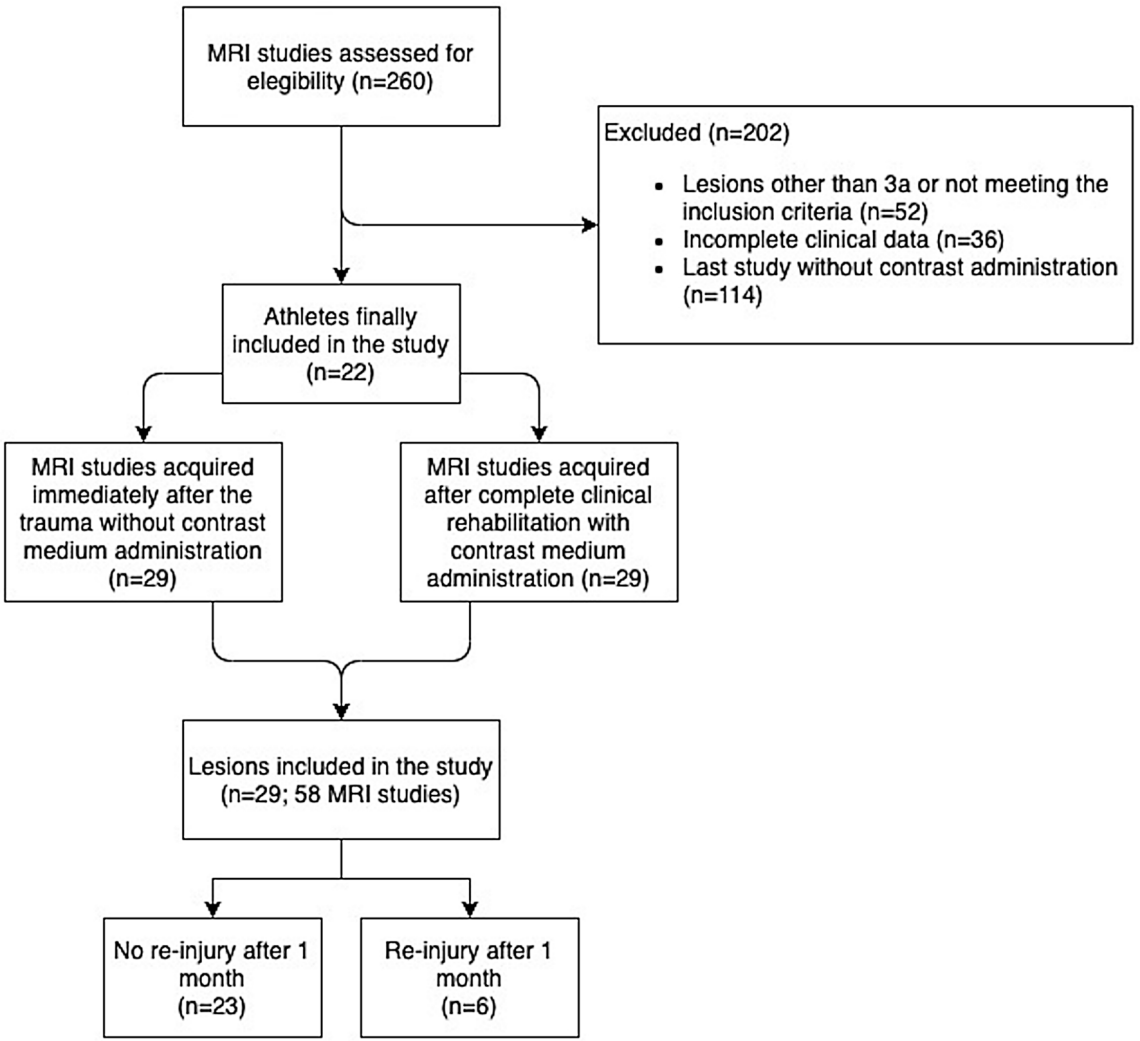
Flowchart showing the selection of records that satisfied the necessary conditions to be included in the study

Of the initial 260 MRI studies, 58 fulfilled the inclusion criteria (Fig. 1). We examined the records of nineteen high-level professional football players and three high-level professional basketball players, some of the athletes had different lesions in various muscles at multiple points in time.

Twenty-nine lesions were finally evaluated. The average age of the selected sample was 27 years (SD ± 5 years; range 20–39). The involved muscles are summarised in Table 3, 79% of the lesions were located at the myotendinous junction while 21% were at the myofascial junction.

**Table 3:** Muscles involved in the injuries

Long adductor, n (%)	1 (3.45)
Biceps femoris, n (%)	10 (34.48)
Lateral gastrocnemius, n (%)	1 (3.45)
Medial gastrocnemius, n (%)	3 (10.34)
Iliopsoas, n (%)	1 (3.45)
Rectus femoris, n (%)	3 (10.34)
Semimembranosus, n (%)	5 (17.24)
Soleus, n (%)	4 (13.79)
Vastus intermedius, n (%)	1 (3.45)

### Lesions’ characteristics

**Fig. 2:**
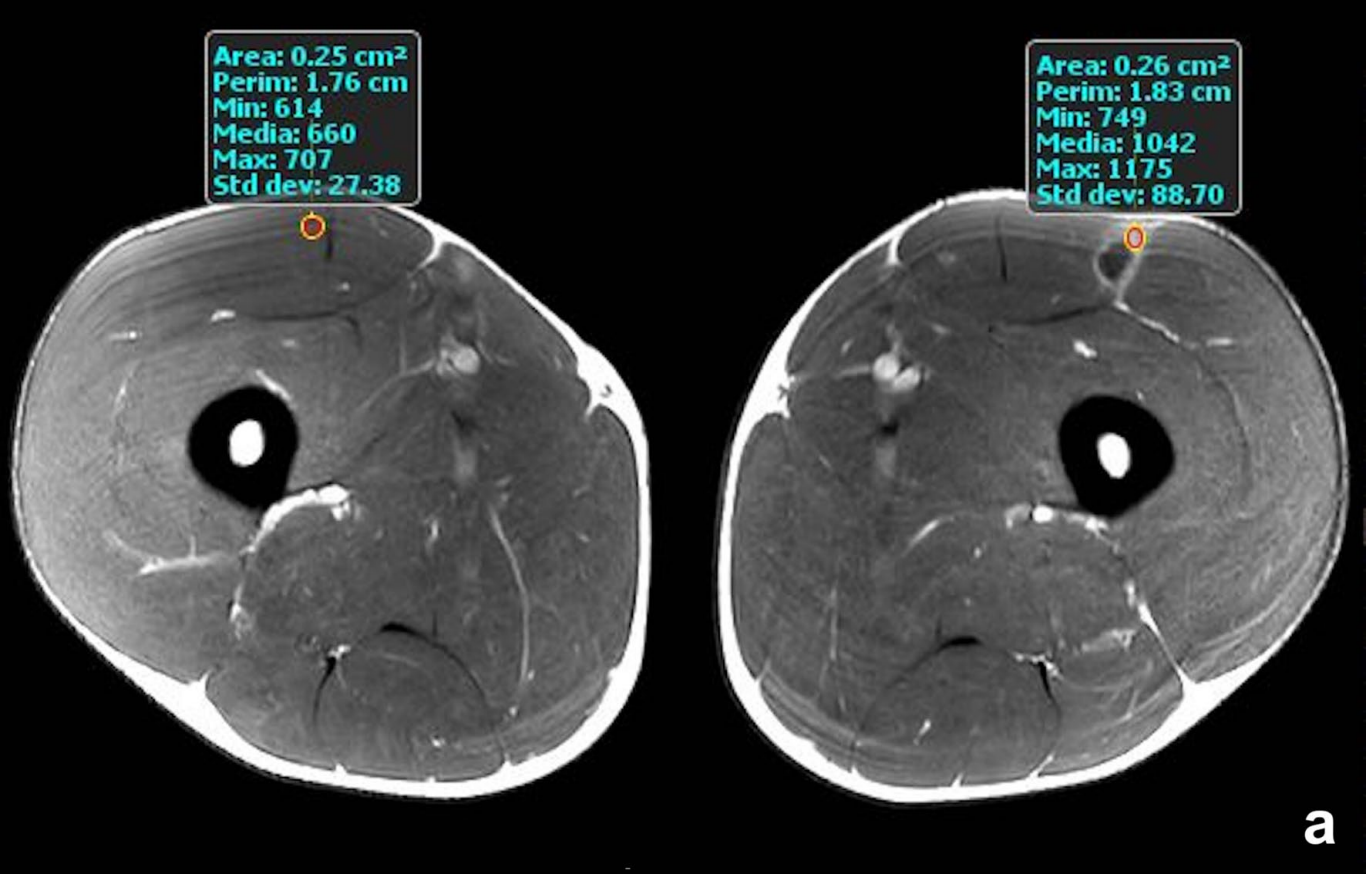
Athlete with a type 3a injury at the myoaponeurotic junction of the rectus femoris muscle. The image was taken several weeks after the injury and shows the persistence of a small intramuscular collection. The scar is not stabilised yet and the use of contrast medium shows a considerable enhancement (MC index = 57.8%)

We obtained an excellent intra-reader reliability with a κ score of 0.97. In 19 cases collections were found in the intrafascial area, along the longitudinal axis of the involved muscles. In the second MRI 13 collections resolved while 6 cystic collections remained (Fig. 2).

The average enhancement value in the lesion areas was 796.6 (SD 257 RANGE 360–1446), while in the healthy muscle areas it was 482 (SD 143.5 RANGE 233–984). The average time from the last contrast-enhanced MRI to return to the field was 11.5 days (SD 8 RANGE 0–31).

In the month following re-entry, 6 athletes (20.6%) developed a re-injury while 23 (79.4%) without re-injury had a return to sport activity.

Players with re-injury resumed high-effort activity after an average of 5.8 days (SD 6.3 RANGE 0–17) from the date of injury; those without re-injury after 13 days (SD 8 RANGE 4–31).

**Fig. 3:**
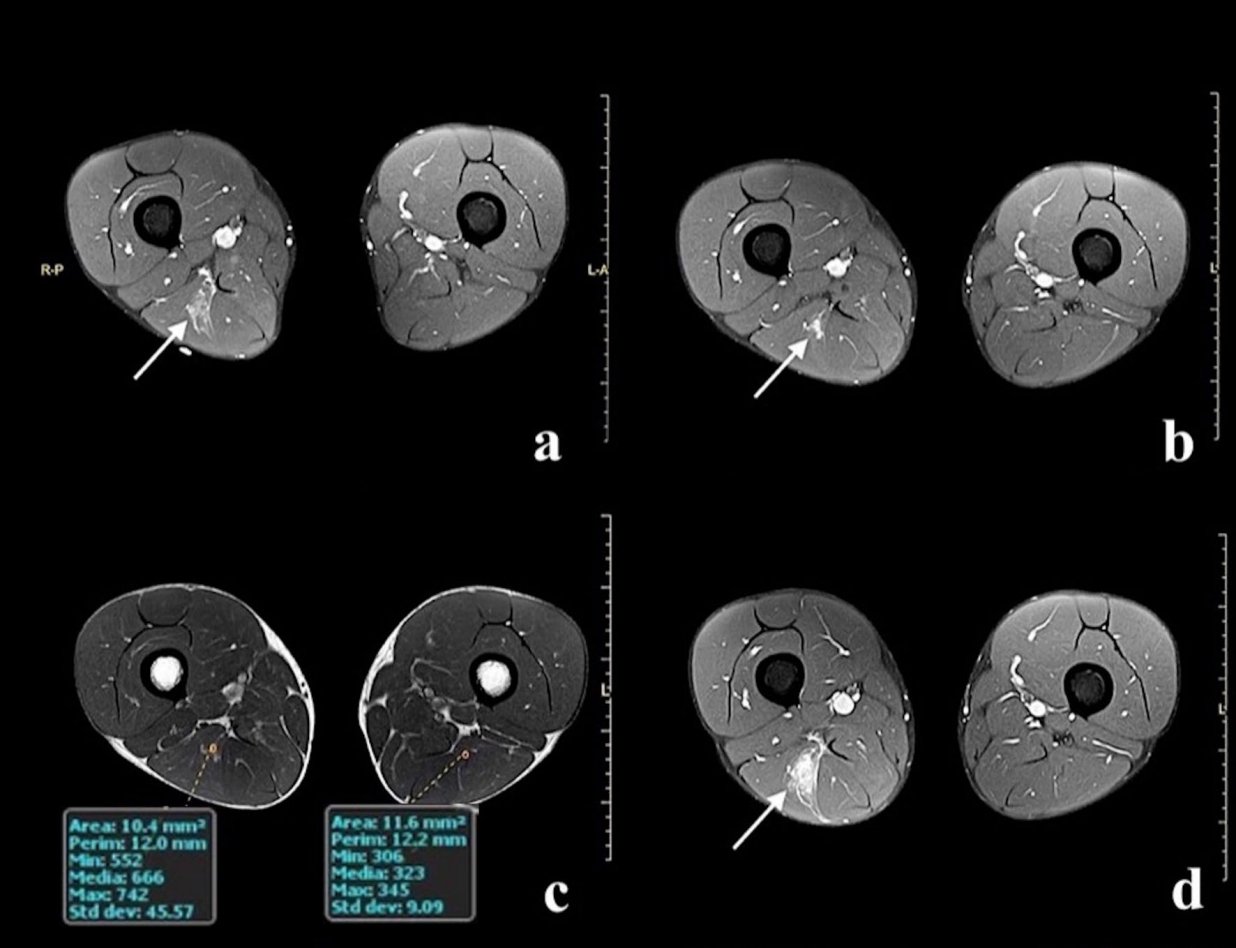
**a** Image obtained with DP TSE sequence with fat suppression shows 3A lesion (white arrow). **b** Symptom resolution check with DP TSE fat suppression sequence, shows effusion and interstitial haemorrhage resorption, residual small area of hyperintense reparative tissue (white arrow). **c** During the same examination in the SE T1 image after contrast medium administration shows MC index 51%. **d** Examination performed after competitive activity recovery; DP TSE fat suppression sequence shows re-injury (white arrow)

**Fig. 4:**
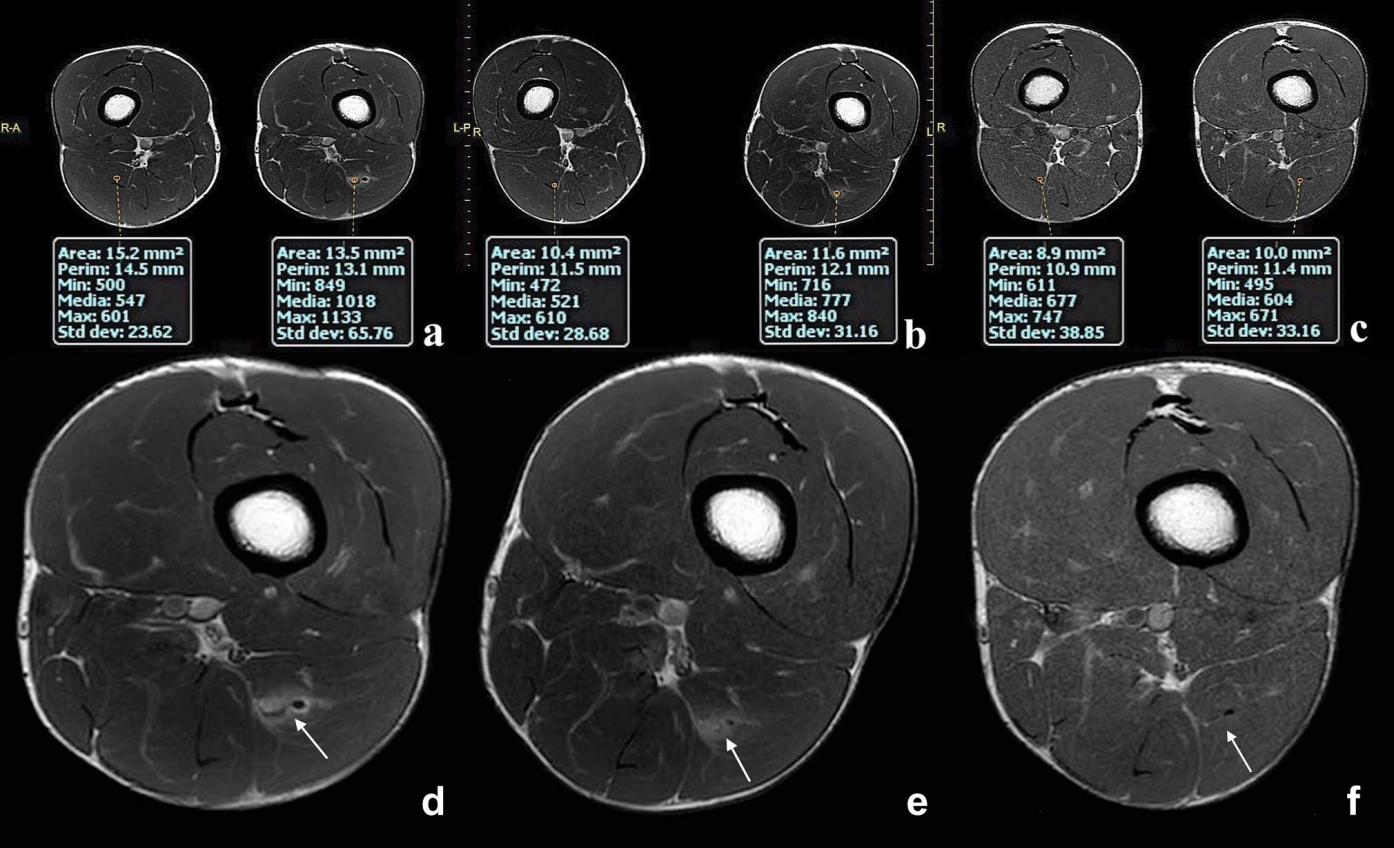
**a** Image obtained after contrast medium administration shows MC index 43%. The image was acquired 14 days after the injury. **d** Magnification of image A at the injury site (white arrow). **b** In the same patient, another image was obtained with the same parameters after contrast medium administration showing MC index reduction to 32%. This image was acquired 21 days after the injury. **e** Magnification of image B at the injury site (white arrow). **c** The same image acquired at the same level after 38 days showed MC index—12% due to fibrous scar formation. **f** Magnification of image C at the injury site (white arrow). Images C and F were obtained two weeks after the player's return to the field, and he did not report any reinjuries

The average MC index value was 47.24 (SD 6.9 RANGE 39.4–54.8) in the group with re-injury (Fig. 3) and 30.1 (SD 16.5 RANGE 0.8–58.1) in the group without re-injury (Fig. 4).

The adjusted values of the MC index in relation to the time elapsed between the last MRI and the actual return to the field were 24.5 (SD 19.4 RANGE 4.4–50.1) in the group with re-injury and 3 (SD 2 RANGE 0.2–8.3) in the group without re-injury, respectively.

The descriptive statistical analysis revealed three MC index values significantly different from the averages of the two samples: one in the group of patients with re-injury, with much lower score (7.08), and two in the group of patients without re-injury, with negative scores (− 2.22; − 25.95). These three measurements were considered as outliers. The sample size was insufficient to obtain a normal distribution. For this reason, data were analysed using the Mann–Whitney and Kruskal–Wallis tests. In both cases, the difference between the mean values of the adjusted MC index in the two groups was considered statistically significant (Z score = − 3.239; H (1, *N* = 28) = 10.6876; *p* of 0.0012 and 0.00108, respectively).

## Discussion

Muscle injuries represent one of the most frequent and most relevant injuries in professional football which is one of the major causes of absence from competition [4, 8, 14–16]. The Union of European Football Associations (UEFA) has expressed its concern over the physical and mental load on modern professional footballers and the possible risk of injury as a result of such loads [1]. Muscle injuries are also common in rugby union [17, 18], Australian Rules football, [19] basketball [20] and other Olympic sports [1, 21]. Muscle injury is not only a problem for the athlete's health but also impacts on the club's performance, the value of the athlete in the sports trade and, indirectly, the value of the whole team. The longer the player's downtime, the greater the economic implications for the sports organisation. For this reason, it is essential to minimise the injury time by finding prognostic factors that can help the clinician to plan the return to the field [22].

Our study, although retrospective, has the advantage of being a single-centre study in which all examinations were read by the same radiologist, resulting in a high degree of reproducibility. An additional strengthening factor in the results' reproducibility was the use of the same sequence and equipment in all studies on which the MC index was then calculated.

In addition, the collected data covers at least ten sporting seasons minimising fluctuations in a club's performance due to external, economic or political factors. Only few published studies have included data from two or more seasons [18–21], and thus, little is known about the natural variations between seasons [7]. In our study, we decided to use the Mueller-Wohlfahrt classification. Several classification systems have been proposed over the years, such as Peetrons, Cohen and O’Donoghue [12]. The Mueller-Wohlfahrt classification, however, is supported by a considerable amount of data and studies have proved its validity for prognostic purposes and its applicability in the clinical-sports context [8].

Furthermore, as our study relies on MRI, the use of classification specifically designed for MRI was deemed to be a thoughtful choice.

In Wangensteen's et al. study [5] it is shown that muscle injuries classified as mild, moderate and severe have quite different healing times. The study shows that mild injuries have a rapid prognosis, while severe injuries have a long lay-off time. The challenge is the grey area in between, where injuries classified as ‘intermediate’ have a very wide range of recovery times, even though they are within the same category [5].

In our study, we decided to include only lesions classified as 3A to reduce the already high degree of variability without focusing on a specific muscle group as in previous studies of Askling et al., Cohen et al. and Ekstrand et al. [4, 16, 23].

**Fig. 5:**
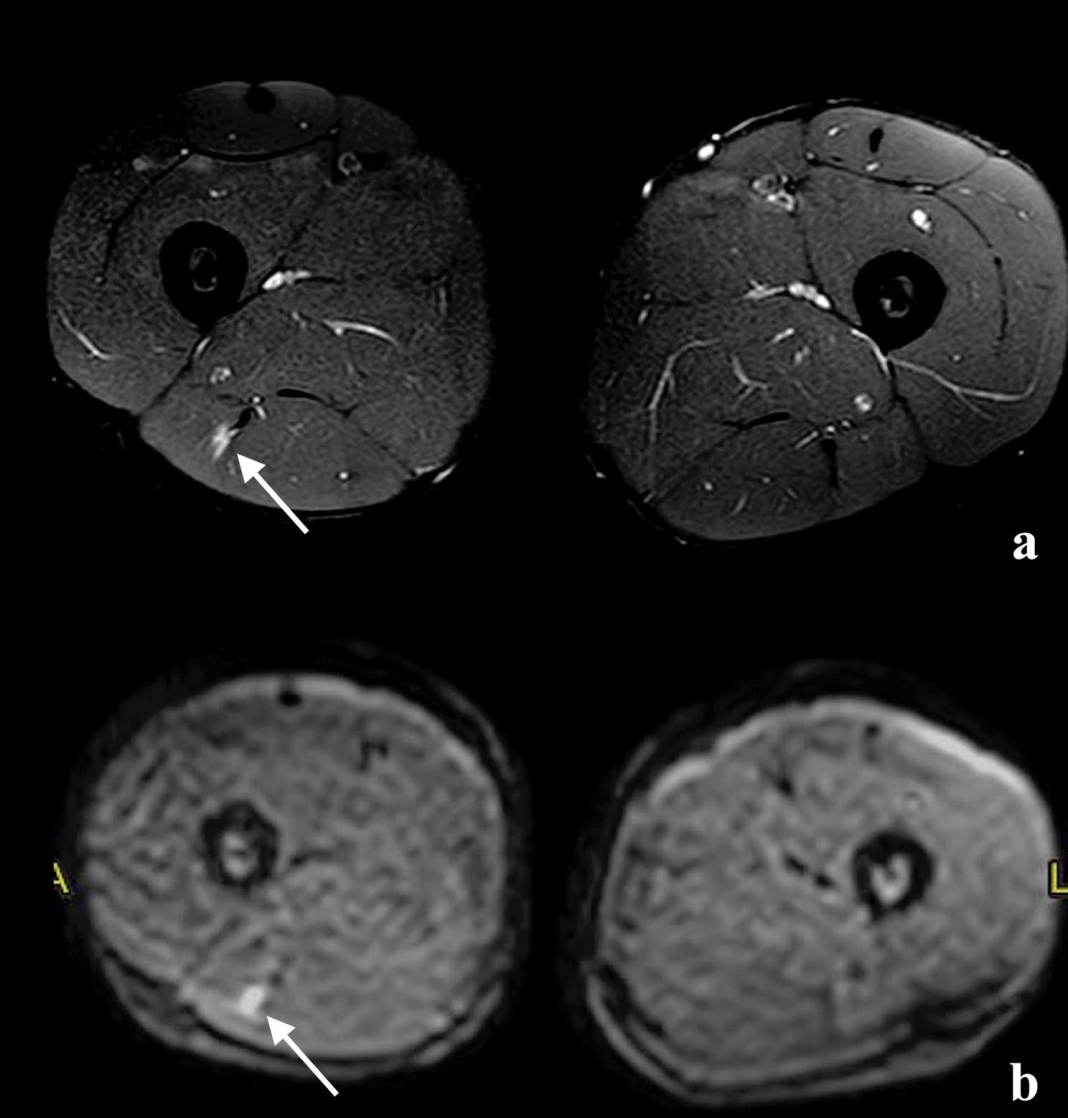
**a** Image obtained with DP TSE sequence with fat suppression shows 3A lesion (white arrow). **b** At the same anatomical region in the DWI study (b = 450), a clear signal hyperintensity is recognisable at the point where the greatest number of muscle fibres were interrupted (white arrow)

The area of injury to be then evaluated with contrast was selected using DWI sequences [3]. A positive association exists between the extent of muscle injury and the average water molecule diffusivity measure in the DWI study [3]. The greater the number of injured fibres, the greater the proportion of free water. All the lesions included in this study showed signal hyperintensity in the long b-value sequences (450; 900), indicating the presence of a sufficient proportion of injured fibres to classify them as type 3a (Fig. 5).

The region of altered DWI signal corresponds to the site of major structural alteration of the muscle fibres and, so, to the site where to assess the cicatricial processes in the healing phase.

Regarding the results obtained, the distribution of injuries recorded is in line with the literature [24].

The distribution of injuries in the muscles themselves was also consistent with other studies in the literature, as was the age distribution of the athletes at the time of injury [25].

The recorded re-injury rate was also only slightly lower (12%) than the average value reported in the literature (16%) but in line with the results reported by Ekstrand et al. [7, 25].

All the athletes underwent contrast-enhanced MRI only after the resolution of symptoms or when clinical evaluation alone was no longer able to discriminate the effective healing status of the injured muscle. An early study would have been of limited use as the individual treatment plan was clinically guided.

As shown in Järvinen's and Dong’s study [26, 27], muscle injuries progress through several healing stages until stiff fibrous scar forms and then stabilises.

The paramagnetic contrast agent accumulates, by definition, in the interstitial space between the cells and, in our case, allows its paramagnetic effect to be evident nearby the forming scar tissue [28].

Based on our results, we believe that the greater the ‘enhancement’ measured with the MC index, the greater the interstitial space not occupied by tissue and, so, the greater the accumulation of contrast medium. This would directly reflect on the maturation phase of the scar, which would assume less and less contrast until it is completely formed and ‘stable’.

Based on the results obtained by comparing athletes with and without re-injury in the month following their return to the field, we can say that an MC index of less than 30% is indicative of good maturation of the scar tissue, while for values above 50% it is prudent not to perform activities with a high functional demand. This assumption, thus, is still empirical and needs to be validated with a prospectively designed study.

The MC index values obtained in the group of athletes with re-injury were significantly higher compared to the control group. The MC index values presented in the results were adjusted to the time elapsed between the last examination and the actual return to the field. The use of an adjusted index allowed more consistent data to be obtained, even if the study was conducted retrospectively.

Being able to discriminate between a ‘stable’ and an ‘unstable’ injury is of fundamental importance in the management of the elite athlete as it would allow precise modulation of recovery times, while reducing the rate of re-injury with enormous tactical and economic advantages for the whole team [29, 30].

In conclusion, we would say that contrast medium in the follow-up of muscular injuries in high-level athletes is certainly a useful tool. The use of the MC index could help the sports physician to guide the rehabilitation of an athlete by adapting the recovery time to the scar stabilisation status as well as to the clinic.

## Limitations and future perspectives

The current study has some limitations. First, it is a retrospective study based on already existent MRI studies, and the RTS time was not directly measured but calculated from the clinical records. Many factors go into an athlete’s return, such as pain threshold, motivation, timing of the season, political/financial factors and, of course, severity of injury. Return to play can be a subjective outcome. Our analysis does not consider any subjective factors associated with the player’s time away from sports. In professional football, where there are a limited number of games and the salaries are high, missed playing time can be costly. There were several circumstances where injuries occurred in preseason and veteran players were rested longer to confirm complete recovery. Conversely, younger, less established players may have returned to play quicker with the aim of achieving a promotion.

Another limitation of the study is the use of contrast agent, which may lead to long-term accumulation in the brain in the case of repeated lesions.

It would be desirable to explore alternative methods that do not involve the use of contrast medium, although, at present, the barrier damage that makes it possible to establish the presence of a mature scar can only be assessed using contrast medium.

The MC index needs to be validated with prospective studies aimed at assessing the rate of recurrence and a possible reduction in the recovery time of athletes. It is in fact possible that, with a greater degree of certainty given by the knowledge of the scar's maturation status, in some cases an early return to play can be achieved. Ideally, a prospective study would predict the RTS time and determine the accuracy of our model.

## Abbreviation

RTS: Return to sport.

## References

[1] Pollock N, James SLJ, Lee JC, Chakraverty R (2014). British athletics muscle injury classification: a new grading system. Br J Sports Med.

[2] Hamilton B, Valle X, Rodas G (2015). Classification and grading of muscle injuries: a narrative review. Br J Sports Med.

[3] Nocerino EA, Aliprandi A, Tavana R (2019). Evaluation of muscle tears in professional athletes using diffusion-weighted imaging and apparent diffusion coefficient: preliminary results. Acta Bio Med Atenei Parmensis.

[4] Cohen SB, Towers JD, Zoga A (2011). Hamstring injuries in professional football players: magnetic resonance imaging correlation with return to play. Sports Health.

[5] Wangensteen A, Guermazi A, Tol JL (2018). New MRI muscle classification systems and associations with return to sport after acute hamstring injuries: a prospective study. Eur Radiol.

[6] Chan O, Del Buono A, Best TM, Maffulli N (2012). Acute muscle strain injuries: a proposed new classification system. Knee Surg Sports Traumatol Arthrosc.

[7] Ekstrand J, Hagglund M, Walden M (2011). Injury incidence and injury patterns in professional football: the UEFA injury study. Br J Sports Med.

[8] Ekstrand J, Askling C, Magnusson H, Mithoefer K (2013). Return to play after thigh muscle injury in elite football players: implementation and validation of the Munich muscle injury classification. Br J Sports Med.

[9] Bryan Dixon J (2009). Gastrocnemius vs. soleus strain: how to differentiate and deal with calf muscle injuries. Curr Rev Musculoskelet Med.

[10] Hancock CR, Sanders TG, Zlatkin MB (2009). Flexor femoris muscle complex: grading systems used to describe the complete spectrum of injury. Clin Imaging.

[11] Kumaravel M, Bawa P, Murai N (2018). Magnetic resonance imaging of muscle injury in elite American football players: predictors for return to play and performance. Eur J Radiol.

[12] Mueller-Wohlfahrt H-W, Haensel L, Mithoefer K (2013). Terminology and classification of muscle injuries in sport: the Munich consensus statement. Br J Sports Med.

[13] von Elm E, Altman DG, Egger M (2007). The Strengthening the Reporting of Observational Studies in Epidemiology (STROBE) statement: guidelines for reporting observational studies. Ann Intern Med.

[14] Bisciotti GN, Volpi P, Alberti G (2019). Italian consensus statement (2020) on return to play after lower limb muscle injury in football (soccer). BMJ Open Sport Exerc Med.

[15] Hall MM (2018). Return to play after thigh muscle injury: utility of serial ultrasound in guiding clinical progression. Curr Sports Med Rep.

[16] Ekstrand J, Healy JC, Waldén M (2012). Hamstring muscle injuries in professional football: the correlation of MRI findings with return to play. Br J Sports Med.

[17] Brooks JHM, Fuller CW, Kemp SPT, Reddin DB (2005). Epidemiology of injuries in English professional rugby union: part 1 match injuries. Br J Sports Med.

[18] Brooks JHM, Fuller CW, Kemp SPT, Reddin DB (2005). Epidemiology of injuries in English professional rugby union: part 2 training Injuries. Br J Sports Med.

[19] Orchard J, Seward H (2002). Epidemiology of injuries in the Australian Football League, seasons 1997–2000. Br J Sports Med.

[20] Meeuwisse WH, Sellmer R, Hagel BE (2003). Rates and risks of injury during intercollegiate basketball. Am J Sports Med.

[21] Engebretsen L, Soligard T, Steffen K (2013). Sports injuries and illnesses during the London Summer Olympic Games 2012. Br J Sports Med.

[22] Chen Y, Buggy C, Kelly S (2019). Winning at all costs: a review of risk-taking behaviour and sporting injury from an occupational safety and health perspective. Sports Med Open.

[23] Askling CM, Tengvar M, Saartok T, Thorstensson A (2007). Acute first-time hamstring strains during high-speed running: a longitudinal study including clinical and magnetic resonance imaging findings. Am J Sports Med.

[24] Hägglund M, Waldén M, Ekstrand J (2013). Risk factors for lower extremity muscle injury in professional soccer: the UEFA Injury Study. Am J Sports Med.

[25] Ekstrand J, Hägglund M, Waldén M (2011). Epidemiology of muscle injuries in professional football (soccer). Am J Sports Med.

[26] Dong Y, Jing Z, Wei L (2013). Magnetic resonance imaging and histopathological analysis of experimental muscle injuries in a rabbit. Biomed Environ Sci.

[27] Järvinen TAH, Järvinen TLN, Kääriäinen M (2005). Muscle injuries: biology and treatment. Am J Sports Med.

[28] Xiao Y-D, Paudel R, Liu J (2016). MRI contrast agents: classification and application (review). Int J Mol Med.

[29] Waldén M, Hägglund M, Ekstrand J (2005). Injuries in Swedish elite football–a prospective study on injury definitions, risk for injury and injury pattern during 2001. Scand J Med Sci Sports.

[30] Waldén M, Hägglund M, Ekstrand J (2005). UEFA Champions League study: a prospective study of injuries in professional football during the 2001–2002 season. Br J Sports Med.

